# Impact of active intra-complex rest intervals on post-back squat versus hip thrust jumping potentiation

**DOI:** 10.1038/s41598-023-47042-7

**Published:** 2023-11-10

**Authors:** Robert Urbański, Piotr Biel, Sebastian Kot, Dawid Perenc, Piotr Aschenbrenner, Petr Stastny, Michał Krzysztofik

**Affiliations:** 1 Department of Biomechanics and Sports Engineering, Gdansk University of Physical Education and Sport, Gdańsk, Poland; 2 Department of Sport and Physical Education, AGH University of Science and Technology, Krakow, Poland; 3 Nutrition and Sports Performance Research Group, The Jerzy Kukuczka Academy of Physical Education in Katowice, 40-065 Katowice, Poland; 4 Department of Sport Games, Faculty of Physical Education and Sport, Charles University in Prague, Prague, Czech Republic; 5 Institute of Sport Sciences, The Jerzy Kukuczka Academy of Physical Education in Katowice, Poland, Mikołowska 72A str., 40-065 Katowice, Poland

**Keywords:** Physiology, Health care

## Abstract

This study investigated the impact of active rest intervals within a lower body complex training session on post-activation performance enhancement (PAPE) response in amateur soccer players. Twelve soccer players took part in four different experimental conditions. These sessions included 2 sets of lower body complex-paired exercises, each involving 3 repetitions of either back squats or hip thrusts at 90% one-repetition maximum (1RM) as a conditioning activity paired with a broad jump and countermovement jump. Between those exercises in active intra-complex rest interval conditions, participants were performing 8 repetitions of bench press at 75%1RM or, in passive intra-complex rest interval conditions, rested while seated. A significant main effect of a set to increase broad jump length (*p* = 0.002), countermovement jump height (*p* = 0.002), and modified reactive strength index (*p* = 0.005) was revealed, without any significant differences between conditions. Post-hoc comparisons showed a significant increase in broad jump length from baseline to Set-2 (231 ± 13 vs. 234 ± 13 cm; *p* = 0.003; ES = 0.22). On the other hand, countermovement jump height and modified reactive strength index significantly increased from baseline to Set-1 (34.4 ± 3.6 vs. 35.6 ± 3.9 cm; *p* = 0.027; ES = 0.31 and 0.4 ± 0.05 vs. 0.45 ± 0.09; *p* = 0.005; ES = 0.66). Results of this study showed that to sustain a high training density, sports practitioners may incorporate upper body exercises within the intra-complex rest interval during lower limb complex training sessions and still elicit a significant PAPE effect.

## Introduction

In the field of sports science, the phenomenon known as post-activation performance enhancement (PAPE) has received considerable attention in last years. PAPE refers to the acute and temporary improvement in athletic performance following a conditioning activity (CA)^1,2^. This unique physiological response has led researchers and athletes alike to explore various strategies for maximizing its benefits^3–6^. Commonly a high-intensity resistance exercise is used as a CA and precedes an explosive exercise with a similar biomechanical movement structure^7^. Research conducted thus far has indicated that the greatest improvement in physical performance occurs approximately between 5 and 7 min after the completion of the CA^8,9^. As the athlete is resting passively during this period, this training method is characterized by low training density and time efficiency^10^. Therefore, this approach may pose practical challenges as athletes have limited time due to other sports commitments, necessitating a highly effective and time-saving training process.

The proposed solution to this problem is to include an exercise engaging a different body part comparing CA and post-CA within the PAPE complex as an active inter-complex rest interval^11–13^. In the case of a lower-limb PAPE complex (e.g., barbell squats [SQ] as the CA and vertical jump as the post-CA), this could be an exercise involving the upper body (e.g., bench press). Considering that the PAPE effect appears to occur locally^2,14,15^, an exercise involving muscle groups other than those primarily involved in the CA and post-CA may not have a substantial effect on its occurrence. To the best knowledge of the authors, only Trybulski et al.^13^ has evaluated the use of an active inter-complex rest interval during an upper-limb PAPE complex. The authors did not show any impact of body-weight Swiss ball leg curls as an active inter-complex rest interval on post-bench press bench press throw potentiation. Insufficient research has been conducted on lower-limb PAPE complexes and the use of high-intensity exercises during active inter-complex rest intervals. Moreover, interestingly, a recent study by Bartolomei et al.^16^ demonstrated that the PAPE effect potentially could occur remotely. Bartolomei et al.^16^ showed an increase in countermovement jump (CMJ) power output after a high-intensity bench press. Theoretically, the non-localized PAPE effect may be associated with an increase in epinephrine and norepinephrine levels following high-intensity exercise^17^. Therefore, in this scenario, implementing a high-intensity upper-body exercise within a PAPE complex may even contribute to enhancing the PAPE effect.

Although the PAPE effect has been studied extensively and it is recommended to couple CA and post-CA in terms of their biomechanical similarity, research on this aspect is still lacking^18,19^. According to this principle, when comparing the effectiveness of inducing the PAPE effect through two different CAs involving the same muscle groups, the one that also requires force production in the same direction as the post-CA may be more efficient. For example, SQ should be more effective for potentiation tasks involving lower limb exercises where force is generated vertically, such as vertical jumping. Conversely, hip thrusts (HT) should be superior for potentiation tasks requiring horizontal force generation, such as broad jumps (BJ) or sprints. Interestingly, Seitz et al.^20^ demonstrated post-SQ BJ potentiation, but the authors did not evaluate the effectiveness of HT as a CA, so there is uncertainty as to whether the effect would be greater after them, considering the principle of similarity. Meanwhile, neither Atalag et al.^18^ nor Carbone et al.^19^ demonstrated a significant improvement in sprint performance after SQ or HT. Despite the fact that during the HT, the generated force vector is comparable to that during sprinting. However, there is a dearth of research comparing the effects of SQ and HT on the PAPE effect in both horizontal and vertical jumps.

Considering the above, by comparing the impact of active and passive intra-complex rest intervals, as well as the alignment of exercises with the dynamic correspondence principle, this study aims to shed light on optimal strategies and examines the trade-off between training density and PAPE magnitude. Therefore, this study examined the effects of bench press exercises implemented within the lower body PAPE complex on the magnitude of this phenomenon, specifically SQ and HT as a CA and CMJ and BJ as post-CA tasks. Moreover, the secondary aim was to compare post-SQ and post-HT vertical and horizontal jumping potentiation. It was hypothesized, that performing bench presses will not affect PAPE response and that SQ will be superior in potentiating CMJ performance while HT in BJ length.

## Results

Reliability results are described in Table 1.

**Table 1:** Intersession reliability and smallest worthwhile change of the analyzed variables.

Variable	ICC (95%CI)	CV (%)	SWC
BJ length	0.85 (0.64 to 0.95)	3.1 ± 2	3 cm
CMJ height	0.96 (0.9 to 0.99)	3.6 ± 1.9	0.7 cm
CMJ relative peak Power	0.97 (0.92 to 0.99)	3.2 ± 1.7	1 W/kg
Contraction Time	0.89 (0.73 to 0.96)	6.4 ± 3.1	24 ms
RSImod	0.91 (0.79 to 0.97)	7.1 ± 2.8	0.02

ICC—intraclass correlation coefficient; CI—confidence interval; CV—coefficient of variation; SWC—smallest worthwhile change; BJ—broad jump; CMJ—countermovement jump; RSImod—modified reactive strength index.

### Broad Jump

Two-way ANOVA did not reveal a statistically significant interaction for BJ length (F = 1.359; *p* = 0.244; η^2^ = 0.11), and no statistically significant main effect of the condition was reported for BJ length (F = 0.351; *p* = 0.623; η^2^ = 0.031). However, a significant main effect of time was found to increase BJ length (F = 8.487; *p* = 0.002; η^2^ = 0.436). Post-hoc comparisons indicated a significant increase in BJ length from baseline to Set-2 (231 ± 13 vs. 234 ± 13 cm; *p* = 0.003; ES = 0.22) (Table 2).

**Table 2:** Comparison of pre- and post-CA jumping performance.

Condition	BA (95%CI)	Set-1 (95%CI)	Set-2 (95%CI)	Effect size		
				BA versus Set-1	BA versus Set-2	Set-1 versus Set-2
Broad jump length [cm]						
SQ-A	231 ± 14 (222 to 240)	232 ± 13 (224 to 241)	234 ± 14 (224 to 243)	0.07	0.21	0.14
SQ-NA	230 ± 14 (221 to 239)	231 ± 15 (222 to 241)	234 ± 11 (227 to 241)	0.07	0.31	0.22
HT-A	232 ± 13 (224 to 240)	234 ± 15 (224 to 243)	232 ± 12 (224 to 239)	0.14	0.00	0.14
HT-NA	231 ± 14 (222 to 240)	236 ± 15 (226 to 245)	238 ± 16 (228 to 248)	0.33	0.45	0.12
Countermovement jump height [cm]						
SQ-A	34.2 ± 3.4 (32 to 36.4)	35.6 ± 4.2 (32.9 to 38.2)	35.4 ± 3.6 (33 to 37.7)	0.35	0.33	0.05
SQ-NA	34.1 ± 3.2 (32.1 to 36.1)	35.9 ± 4 (33.4 to 38.4)	35.5 ± 3.8 (33.1 to 37.9)	0.48	0.38	0.1
HT-A	34.5 ± 4.1 (31.9 to 37.1)	35.4 ± 3.7 (33.1 to 37.7)	35.7 ± 3.5 (33.4 to 37.9)	0.22	0.3	0.08
HT-NA	35.1 ± 4.1 (32.4 to 37.6)	35.7 ± 4.1 (33.1 to 38.3)	35.6 ± 3.4 (33.4 to 37.8)	0.14	0.13	0.03
Countermovement jump relative peak power [W/kg]						
SQ-A	51.3 ± 5.7 (47.7 to 54.9)	53.1 ± 4.8 (50.1 to 56.2)	52 ± 4.1 (49.4 to 54.6)	0.33	0.14	0.24
SQ-NA	51.6 ± 5.7 (48 to 55.3)	53.7 ± 4.8 (50.7 to 56.8)	53.3 ± 4.4 (50.6 to 56.1)	0.38	0.32	0.08
HT-A	51.6 ± 5.6 (49.1 to 55.1)	51.6 ± 4.4 (48.8 to 54.4)	52.6 ± 4.6 (49.7 to 55.6)	0.00	0.19	0.21
HT-NA	52.2 ± 4.2 (49.5 to 54.9)	52.7 ± 5.2 (49.3 to 56)	52.4 ± 4.9 (49.3 to 55.5)	0.1	0.04	0.06
Countermovement jump contraction time [ms]						
SQ-A	875 ± 99 (812 to 937)	798 ± 157 (698 to 898)	841 ± 175 (730 to 952)	0.57	0.23	0.25
SQ-NA	833 ± 106 (766 to 901)	771 ± 141 (682 to 861)	746 ± 130 (663 to 828)	0.48	0.71	0.18
HT-A	852 ± 72 (807 to 898)	852 ± 60 (814 to 890)	870 ± 60 (832 to 908)	0.00	0.26	0.29
HT-NA	898 ± 148 (804 to 992)	838 ± 122 (761 to 916)	835 ± 84 (782 to 889)	0.43	0.51	0.03
Modified reactive strength index						
SQ-A	0.39 ± 0.06 (0.36 to 0.44)	0.47 ± 0.12 (0.39 to 0.54)	0.44 ± 0.11 (0.37 to 0.51)	0.81	0.54	0.25
SQ-NA	0.41 ± 0.05 (0.38 to 0.45)	0.48 ± 0.1 (0.4 to 0.56)	0.49 ± 0.11 (0.42 to 0.56)	0.85	0.9	0.09
HT-A	0.41 ± 0.04 (0.38 to 0.43)	0.42 ± 0.04 (0.39 to 0.44)	0.41 ± 0.04 (0.39 to 0.43)	0.24	0.00	0.24
HT-NA	0.4 ± 0.06 (0.36 to 0.44)	0.43 ± 0.07 (0.39 to 0.48)	0.43 ± 0.06 (0.39 to 0.47)	0.44	0.48	0.00

BA—baseline; CA—conditioning activity; CI—confidence interval; SQ-A—squat conditioning with an active rest interval; SQ- NA—squat conditioning without an active rest interval; HT-A—hip thrust conditioning with an active rest interval; HT-NA—hip thrust conditioning without an active rest interval.

### Countermovement jump

Two-way ANOVA didn’t show statistically significant interactions for CMJ_JH_ (F = 0.609; *p* = 0.722; η^2^ = 0.052), CMJ_PP_ (F = 1.631; *p* = 0.153; η^2^ = 0.129), CMJ_CT_ (F = 1.772; *p* = 0.118; η^2^ = 0.139), and RSImod (F = 1.865; *p* = 0.154; η^2^ = 0.145). Similarly, no statistically significant main effect of the condition was reported for CMJ_JH_ (F = 0.123; *p* = 0.946; η^2^ = 0.011), CMJ_PP_ (F = 0.988; *p* = 0.374; η^2^ = 0.082), CMJ_CT_ (F = 2.811; *p* = 0.055; η^2^ = 0.204), and RSImod (F = 2.426; *p* = 0.83; η^2^ = 0.181). Moreover, no significant main effect of time for CMJ_PP_ (F = 1.853; *p* = 0.18; η^2^ = 0.144) and CMJ_CT_ (F = 3.962; *p* = 0.062; η^2^ = 0.265) was reported. However, a significant main effect of time to increase CMJ_JH_ (F = 8.487; *p* = 0.002; η^2^ = 0.436), and RSImod (F = 9.568; *p* = 0.005; η^2^ = 0.465) was revealed. Post-hoc comparisons showed a significant increase in CMJ_JH_ (34.4 ± 3.6 vs. 35.6 ± 3.9 cm; *p* = 0.027; ES = 0.31) and RSImod from baseline to Set-1 (0.4 ± 0.05 vs. 0.45 ± 0.09; *p* = 0.005; ES = 0.66) (Table 2).

## Discussion

The main objective of this study was to determine the effects of active intra-complex rest intervals inside the lower-body PAPE complex on the occurrence and magnitude of this effect, as well as to compare post-SQ and post-HT CMJ and BJ potentiation in soccer players. This study did not reveal any significant differences in the lower limb PAPE effect between active intra-complex rest interval compared to passive intra-complex rest interval. Similarly, there were no significant differences in the CMJ and BJ enhancements, regardless of whether SQ or HT was used as the CA. Although the differences were not significant, it should be emphasized that the magnitude of the PAPE effect was greater when passive intra-complex rest interval was employed, and when the exercises within the complex were aligned with the dynamic correspondence principle (i.e., HT paired with BJ and SQ paired with CMJ). Therefore, for maximizing the lower limb PAPE effect, we recommend the use of passive intra-complex rest interval. On the other hand, to maintain high training density, sports practitioners may also incorporate upper body exercises within the active intra-complex rest interval during lower limb PAPE. This approach can be utilized, for instance, during periods when a high training volume is required, such as in the preseason when the emphasis is placed on resistance training, or in situations where the available time for training is limited. However, they should be aware that the PAPE magnitude may be reduced.

Alternating sets of resistance exercises involving different parts of the body (i.e., lower and upper body muscles) have been used in resistance training for a long time and have been identified as a successful method to increase training density without impacting athletic performance^13,25–27^. Although a complex training method is well studied and its low time efficiency due to the need for long rest periods after the CA (4–8 min) has been indicated as their disadvantage, not much research has been done that has looked for solutions to this drawback^11–13^. To the best of the authors' knowledge, this is the second study yet that has evaluated the effect of employing an active intra-complex rest interval during PAPE complexes^13^, and probably the first in the case of the lower limbs. Study by Trybulski et al.^13^ did not show an impact of applying active intra-complex rest intervals on the upper-body PAPE effect magnitude. Similarly, this study's findings revealed no differences between the examined conditions. Nonetheless, it should be noted that although statistically insignificant, larger effect sizes were observed in the conditions with passive intra-complex rest intervals for certain variables. Specifically, the enhancement in BJ length after HT-NA was moderate (g = 0.33 in Set-1 and g = 0.45 in Set-2 compared to baseline), whereas after HT-A it was either small (g = 0.14 in Set-1) or not observed (g = 0.00 in Set-2). Similarly, regarding the RSImod in the SQ-NA condition, the enhancement in Set-2 was large (g = 0.9) and moderate in the SQ-A condition (g = 0.54). These disparities between the current study and the study by Trybulski et al.^13^ may have been influenced by the fact that Trybulski et al.^13^ applied velocity loss control during CA, individually adjusting its volume and controlling the level of induced fatigue. In contrast, the CA in this investigation consisted of constant CA variables through sets of 3 repetitions at 90% 1RM. Despite the growing popularity of velocity-based training, velocity-controlling devices are still not widely used, so we chose this protocol to enhance the ecological value of this study. In conclusion, the results of this study and the study by Trybulski et al.^13^ indicates that applying active intra-complex rest intervals in the form of exercises targeting a distinct body region within the PAPE complex still allows for benefiting from the PAPE effect.

As stated previously, there is a lack of investigations on the utilization of active intra-complex rest intervals in PAPE complexes; however, there are studies evaluating the local and non-local occurrence of this phenomenon^14,16^. On one hand, the PAPE effect seems to have a local nature and is associated with mechanisms such as increased muscle temperature or muscle fiber water content in the exercised muscle groups^2^. On the other hand, the non-local PAPE effect may be explained by the elevation of catecholamine concentration due to high-intensity CA^17^. In this scenario, for instance, the combination of SQ or HT as a CA and bench press during active-intra complex rest interval, as in this study, theoretically may even induce a greater PAPE effect in subsequently performed jumps. However, this wasn't the case in this study. The results on this matter are contradictory; for example, Cuenca-Fernandez et al.^14^ did not reveal significant differences in the effectiveness of three different CAs, namely, 4 repetitions of SQ, 4 repetitions of bench press, and a combination of both, in eliciting the PAPE effect assessed by squat jumps. Nevertheless, similarly to the findings of this study, Cuenca-Fernandez et al.^14^ noted a lower magnitude of the PAPE effect in protocols involving bench press. In contrast, Bartolomei et al.^16^ demonstrated improved CMJ absolute power output after 5 sets of a single bench press repetition at 90% 1RM. However, it is important to emphasize that in the Bartolomei et al.^16^ study, participants performed CMJ only at the 12th and 14th minutes after the CA, whereas in this study and the study by Cuenca-Fernandez et al. (2017) participants performed CMJ at the 6th, 5th and 8th minute, respectively. Furthermore, the volume and intensity scheme used for bench press in this study did not align with the guidelines to induce the PAPE effect and differed significantly (single set of 8 repetitions at 75%1RM vs. 5 sets of single repetitions at 90%1RM) from those employed by Bartolomei et al.^16^. Interestingly, another study by Bartolomei et al.^25^ also demonstrated a significant increase in absolute CMJ power output after bench press (using the same conditioning activity as in their other study^16^). However, no such effect was reported in the bench press throw after the same back squat protocol (5 sets of a single repetition at 90%1RM). Therefore, it appears that the local PAPE effect may be linked to the amount of muscle mass involved in the conditioning activity, suggesting that the non-localized effect is also associated with it^25,26^. During the active inter-complex rest interval conditions, both BS and HT as well as bench presses were utilized, resulting in a larger muscle area being engaged compared to Bartolomei et al.^16,25^ studies. This could potentially explain why the PAPE effect was attenuated. However, it is worth noting that a non-local PAPE effect might become evident in later time frames than the local PAPE effect or if different bench press variables were utilized.

Studies directly comparing SQ vs. HT as CAs on the PAPE phenomenon are limited to evaluating their impact on sprint performance and vertical jumping^18,19^. However, there is a lack of information on whether the PAPE effect induced by these exercises as CAs would differ for vertical and horizontal jumping performance. One of the fundamental principles of PAPE suggests that the CA and post-CA should have a similar movement structure, considering the range of motion, muscle activity, and force production vector^27,28^. This study found no significant differences in the CMJ and BJ enhancements, regardless of whether SQ or HT was used as the CA. Although, similarly to the type of intra-complex rest intervals, the effect size was insignificantly larger when HT-NA was used before BJ compared to SQ-NA (g = 0.33–0.45 vs. g = 0.07–0.31), and conversely, SQ-NA before CMJ (g = 0.38–0.48) compared to HT-NA (g = 0.13–0.14). The observed patterns suggest that a principle of similarity between CA and post-CA may exist. Because HT requires more hip extension, and force expression remains high as the hip approaches full extension^29–31^, it appears that HT may be more specific for tasks that require high force expression, such as when the hip nears full extension (as in a BJ). However, the modest results obtained in this study do not strongly support this hypothesis. Further research is needed to clarify the relationship between exercise specificity and force production for optimal utilization of the post-activation potentiation effect.

Despite providing valuable practical insights for athletes and instructors utilizing the PAPE effect in their training, this study should be evaluated in light of its limitations. First, the PAPE effect was only evaluated over two sets of complexes in this study's protocol, so it is unknown how athletic performance would change over consecutive sets. In addition, this study did not include physiological measurements or biomechanical analyses (such as comparing a hip joint range of motion during SQ, HT vs. CMJ and BJ), so we cannot precisely explain why these results were observed. The participants in our study were moderately trained soccer players; therefore, extending these results to other groups should be done with caution. This study highlights the need for future research on the application of active intra-complex rest intervals in PAPE complexes, including implementation over long-term interventions. In addition, it emphasizes the importance of investigating the non-local PAPE effect along with establishing its underlying mechanisms.

## Conclusions

This study did not reveal any statistically significant differences in the lower body PAPE effect when comparing an active intra-complex rest interval to a passive intra-complex rest interval. Likewise, there were no significant variations in CMJ and BJ enhancement, regardless of whether SQ or HT was applied as the conditioning activity. While the differences were not statistically significant, it is important to highlight that the magnitude-based effect sizes were greater when a passive intra-complex rest interval was employed and when the exercises within the complex were aligned with the dynamic correspondence principle (e.g., hip thrust paired with BJ, and back squat paired with CMJ). Consequently, for optimizing the lower limb PAPE effect, employing a passive intra-complex rest interval and pairing CA and post-CA exercises according to the dynamic correspondence principle is recommended. On the other hand, to sustain a high training density, sports practitioners may also incorporate upper body exercises within the intra-complex rest interval during lower limb PAPE. Nonetheless, they should be aware that the PAPE magnitude could potentially diminish.

## Material and methods

### Experimental approach to the problem

**Figure 1:**
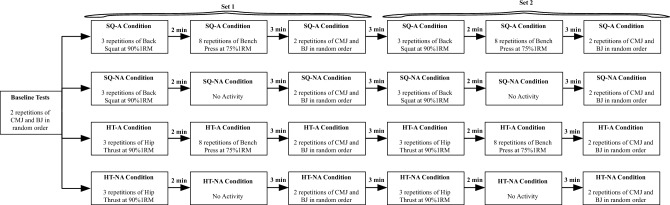
Schematic representation of the experimental sessions. CMJ—countermovement jump; BJ—broad jump; SQ-A—squat conditioning with an active rest interval; SQ- NA—squat conditioning without an active rest interval; HT-A—hip thrust conditioning with an active rest interval; HT-NA—hip thrust conditioning without an active rest interval; 1RM—one-repetition maximum.

To describe the influence of intra-complex active recovery on lower body PAPE effect, cross-sectional randomized research consisted of one familiarization and four experimental sessions were conducted. During the familiarization session, a one-repetition maximum (1RM) test was conducted for the SQ, HT and bench press exercises. In the experimental sessions, participants performed a baseline measurement of the CMJ and BJ performance. They then engaged in a CA consisting of 3 repetitions of SQ or HT at 90% of their 1RM. In the case of sessions with an active intra-complex rest interval, participants performed bench presses (SQ-A and HT-A) or rested in a seated position during sessions without an active intra-complex rest interval (SQ-NA and HT-NA). Finally, participants repeated the CMJ and BJ exercises with 2 repetitions each. In total, participants completed two sets of each complex (Fig. 1).

### Participants

Twelve amateur soccer players (age range: 18–19 years; body mass: 69.8 ± 6.6 kg; height: 175 ± 8 cm; SQ 1RM: 108 ± 13 kg) participated in the experiment. The study took place at the end of the pre-season period, and the participants additionally took part in 3 soccer training sessions a week. The following criteria were used to select participants for the study: (i) absence of neuromuscular and musculoskeletal disorders, (ii) a minimum of two years of experience in resistance training, and (iii) regular participation in soccer and resistance training for at least one year prior to the study. For the duration of the study, participants were instructed to maintain their typical dietary and sleep habits and to refrain from consuming stimulants and alcoholic beverages. In addition, they were instructed to refrain from performing additional resistance exercises within 48 h of the examination to prevent fatigue. Participants were free to withdraw from the experiment at any moment, and they were provided with full details on the potential risks and benefits of the study before providing written informed consent. However, the participants were not told what the expected results of this study were. The study protocol was approved by the Bioethics Committee for Scientific Research, at the Academy of Physical Education in Katowice, Poland (3/2021) and adhered to the ethical standards specified in the 2013 Helsinki Declaration.

A sample size estimation using G*Power software (version 3.1.9.2, Dusseldorf, Germany) showed that to provide 80% power with a significance level of 0.05, correlation among repeated measures of 0.5 in this study design (n = 12; two-way repeated-measures ANOVA) an effect size of at least g = 0.39 will be required. This effect size falls within values reported in previous PAPE studies with similar training settings, namely high-intensity back squats as a complex activator on subsequent vertical and horizontal jumping performance^32–34^, with a 4–8-min intra-complex rest interval^35^.

### Familiarization session

The session began with a general warm-up consisting of 5 min of moderate-intensity stationary cycling, which was followed by dynamic stretching exercises targeting both single- and multi-joint movements, as well as 2 CMJ and 2 BJ performed at 70% perceived effort. Subsequently, participants underwent SQ or machine HT (in random order), separated by bench press 1RM testing. Exercises were then performed with sets of 10, 6, 4, and 3 repetitions using 20%, 40%, 60%, and 80%, respectively, of the participant's self-estimated 1RM. For each successive attempt, the load was gradually increased by 2.5 to 5 kg. Participants' attempts were classified as unsuccessful if they were unable to attain parallel (considered as a top of thigh) depth in the squat position or in the case of hip thrusts fully extended hips. For the bench press, participants had to touch the chest and fully extend their elbows. The greatest weight that could be lifted without assistance was recorded as the 1RM. All participants obtained their 1RM values within a maximum of five attempts, with five-minute recovery intervals between each attempt. In addition, each participant was given details on the forthcoming experimental sessions, which included CMJ and BJ performance assessments.

### Experimental session

After a similar warm-up as during the familiarization session, the participants performed a set at 50%1RM for 6 repetitions, and the second set at 70%1RM for 3 repetitions of either SQ or HT (depending on which exercise was performed later) and bench press. Approximately, 5 min later a CMJ and BJ baseline tests (in random order) started. The CA began after an additional 5 min. Depending on the condition, the CA consisted of 3 repetitions of SQ or HT with loads corresponding to 90%1RM. After completing the CA, participants performed 8 repetitions of bench press at 75%1RM (because this repetitions and intensity range falls within the recommended muscle hypertrophy zone^36^) approximately 2 min later. Three minutes later, participants performed two repetitions of CMJ and BJ in random order, followed by a 3-min rest, before undertaking the next complex. During the session without an active rest period, participants rested in a seated position instead of performing bench presses.

The experimental sessions were separated by a 3–4 day rest period. Participants were instructed to skip heavy lower-body training during the whole study.

### Countermovement jump assessment

The performance of CMJ was assessed using a dual force plate system with a sampling rate of 1000 Hz (Force Decks, Vald Performance, Australia), a validated and reliable device for measuring vertical jump kinematics^37^. Prior to the jump, participants were instructed to stand still for the initial 2-3 s for data collection allowing for body mass determination. To minimize arm swing during the jump, each participant was instructed to keep their arms on their hips throughout the jump. Then, they were instructed to execute a downward movement to a depth of their choosing and "jump as high as possible". Participants were instructed to land in the same position as the take-off in the mid-section of the force plate, and the jump was considered invalid if the athlete landed behind the force plate. Participants returned to the starting position after each jump and repeated the procedure twice for a total of two jumps, with a 30 s rest interval between attempts. The following parameters, including jump height, relative peak power, modified reactive strength index, and contraction time were evaluated. The jumping height was determined by using the following equation:

$$Jump\,height = \frac{1}{2} \cdot \left( {TOV^{2} /g} \right).$$

where: TOV—vertical velocity of the center of mass at take-off; $g=9.81 m\cdot {sec}^{-2}$  

The RSImod was calculated by dividing jump height by contraction time (in seconds). Contraction time was considered as the duration of time between the initiation of the countermovement and take-off. The initiation of movement was defined as the point when the total vertical ground reaction force deviated -20 N from body mass, and take-off was set to the point when the total vertical ground reaction force dropped below 10 N. Contraction time quantified the entire movement time from initiation of movement to take-off. The greatest jump height recorded was chosen for further analysis.

### Broad jump assessment

Participants positioned themselves at the starting line, aligning their legs in a parallel manner and positioning their feet at a distance equivalent to the width of their shoulders. Afterwards, they received instructions to bend their knees (choosing the depth of the bend themselves) and place their arms behind their torso. Following this, they generated a powerful thrust by extending their legs, propelling their arms forward, and executing a maximal jump for distance. The measurement of the distance jumped from the start line to the closest heel, in centimetres, was conducted by the same researcher. Each participant undertook two attempts with a rest interval of 30 s between each jump. A better attempt was retained for subsequent analysis.

### Statistical analysis

All statistical analyses were performed using SPSS (version 25.0; SPSS, Inc., Chicago, IL) and were shown as mean with standard deviation (± SD). Statistical significance was set at *p* < 0.05. The normality of data distribution was checked using Shapiro–Wilk tests.

The relative (2-way mixed effects, absolute agreement, and single-rater intraclass correlation coefficient) and absolute (coefficient of variation) reliability were calculated. The thresholds for interpreting intraclass correlation coefficient results were: 0.5 “poor,” 0.5–0.75 “moderate”, 0.76–0.9 “good,” and 0.0.90 as “excellent”^38^. Although for a coefficient of variation results were, 10% “very good,” 10–20% “good,”, 21–30% “acceptable,” and 0.30% “not acceptable”^39^. The 2-way ANOVAs (4 [SQ; SQ-A; HT; HT-A] 3-time points [BA; Set-1, Set-2]) were used to investigate the influence of CA and active intra-complex rest intervals on CMJ and BJ performance. When a significant main effect or interaction was found, the post hoc tests with Bonferroni correction were used to analyze the pairwise comparisons. The magnitude of mean differences was expressed with standardized effect sizes. Thresholds for qualitative descriptors of Hedge’s g was interpreted as < 0.20 “small,” 0.21–0.79 “medium,” and 0.0.80 as “large.”^40^ The smallest worthwhile change (SWC, calculated using formula 0.2 × test-values standard deviation)^41^ was used to define whether revealed differences are practically meaningful.

## Data Availability

The datasets used and analysed during the current study are available from the corresponding author on reasonable request.
